# ATP-dependent motor activity of the transcription termination factor Rho from *Mycobacterium tuberculosis*

**DOI:** 10.1093/nar/gkv505

**Published:** 2015-05-20

**Authors:** François D'Heygère, Annie Schwartz, Franck Coste, Bertrand Castaing, Marc Boudvillain

**Affiliations:** 1 Centre de Biophysique Moléculaire, CNRS UPR4301, rue Charles Sadron, 45071 Orléans cedex 2, France; 2 Ecole doctorale Santé, Sciences Biologiques et Chimie du Vivant (ED 549), Université d'Orléans, Orléans, France; 3 ITP Sciences Biologiques & Chimie du Vivant, Université d'Orléans, Orléans, France

## Abstract

The bacterial transcription termination factor Rho—a ring-shaped molecular motor displaying directional, ATP-dependent RNA helicase/translocase activity—is an interesting therapeutic target. Recently, Rho from *Mycobacterium tuberculosis* (_Mtb_Rho) has been proposed to operate by a mechanism uncoupled from molecular motor action, suggesting that the manner used by Rho to dissociate transcriptional complexes is not conserved throughout the bacterial kingdom. Here, however, we demonstrate that _Mtb_Rho is a *bona fide* molecular motor and directional helicase which requires a catalytic site competent for ATP hydrolysis to disrupt RNA duplexes or transcription elongation complexes. Moreover, we show that idiosyncratic features of the _Mtb_Rho enzyme are conferred by a large, hydrophilic insertion in its N-terminal ‘RNA binding’ domain and by a non-canonical R-loop residue in its C-terminal ‘motor’ domain. We also show that the ‘motor’ domain of _Mtb_Rho has a low apparent affinity for the Rho inhibitor bicyclomycin, thereby contributing to explain why *M. tuberculosis* is resistant to this drug. Overall, our findings support that, in spite of adjustments of the Rho motor to specific traits of its hosting bacterium, the basic principles of Rho action are conserved across species and could thus constitute pertinent screening criteria in high-throughput searches of new Rho inhibitors.

## INTRODUCTION

RNA helicases are ubiquitous motor proteins that remodel RNA and RNA-protein structures and are involved in all steps of RNA metabolism (1,2). Their activities are often implicated in infection and disease and thus constitute attractive prognostic/diagnostic markers or potential drug targets (3). RNA helicases generally rely on one of two distinct mechanisms of action (1,4). Some RNA helicases are directional, NTP-fueled translocase enzymes exhibiting some degree of processivity. In this case, the helicase loads onto a single-stranded RNA segment and then uses the energy derived from ATP hydrolysis to migrate toward one end of the RNA chain, removing bound obstacles (such as a complementary strand) from its path. This mechanism is reminiscent of that used by DNA helicases (5). Other RNA helicases are local ‘disruptases’ that load on RNA and remodel/disrupt its local structure (or local interaction with an RNA-binding protein) in an ATP-dependent fashion. In this case, the RNA helicase acts as a non-directional and non-processive molecular switch. Local ‘disruptases’ are best exemplified by DEAD-box proteins which compose the largest group of RNA helicases. Upon ATP binding, DEAD-box proteins adopt a tight RNA-bound, ‘close’ state able to denature local RNA structures; upon release of the products of ATP hydrolysis, they revert to an RNA-free ‘open’ state allowing enzyme recycling (1,6).

**Figure 1.**
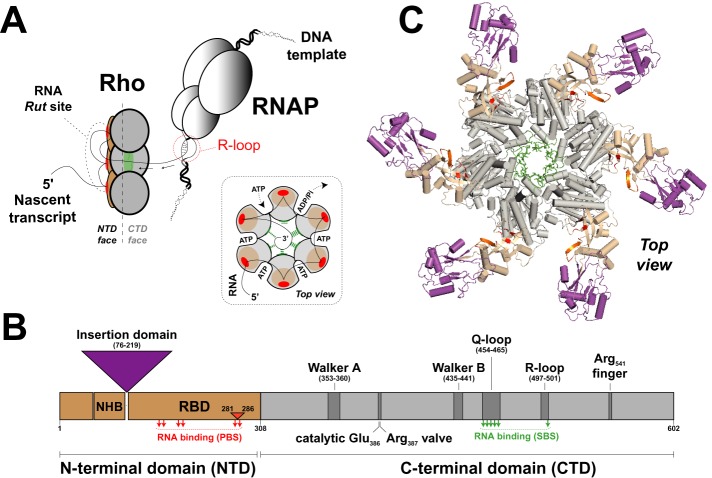
Transcription termination factor Rho. (**A**) A model of Rho-dependent transcription termination based on studies of _Ec_Rho (7). Once bound to a C-rich *Rut* site, the Rho hexamer translocates along the nascent transcript in an ATP-dependent fashion until it catches up with RNAP and triggers dissociation of the TEC. Obstacles such as transcriptional R-loops are removed in the process. Direct contacts between Rho and RNAP, which may exist prior to Rho binding to the nascent transcript (55), are not represented here. Similarly, the exact pathway leading to TEC dissociation once Rho has caught up with the RNAP is still debated (7,8) and is not detailed here. A schematic of the RNA-bound Rho hexamer during ‘tethered tracking’ is also shown in the inset. (**B**) Diagram showing the arrangement of key motifs within the primary sequence of Rho subunits. The N-terminal helix bundle (NHB) domain may contribute to RNA recruitment to the Rho hexamer (36) which is ensured primarily by residues within the RNA binding domain (RBD). The large insertion domain present in _Mtb_Rho (13) is depicted by a purple triangle. Amino acid numbering corresponds to the _Mtb_Rho protein. PBS and SBS residues contacting RNA in _Ec_Rho structures (10,37) are depicted by red and green arrows, respectively. The 6 aa insert between PBS residues of _Mtb_Rho is depicted by a small orange triangle. (**C**) A model of the _Mtb_Rho hexamer. An homology model of the _Mtb_Rho subunit was built with Swiss-Model (http://www.swissmodel.expasy.org/) without pre-defined template. Then, the _Mtb_Rho hexamer was reconstructed by aligning modeled _Mtb_Rho subunits with subunits of the _Ec_Rho hexamer (PDB: 3ICE) using Pymol software (http://www.pymol.org). Apart from the insertion domain, the _Mtb_Rho and _Ec_Rho subunits are structurally similar (rmsd ∼ 1.3 Å). Color coding is the same as in panel B.

The bacterial transcription termination factor Rho is an important model of ring-shaped helicase (4,5). Rho unwinds deleterious RNA:DNA duplexes (R-loops) that are formed behind RNA polymerase (RNAP) during transcription and dissociates transcription elongation complexes (TECs) at specific loci of bacterial genomes (7,8). The molecular mechanisms used by Rho to unwind duplexes and dissociate TECs have been studied extensively, although work has focused primarily on the Rho factor from *Escherichia coli* (_Ec_Rho) (7,9). These studies revealed that Rho uses a sophisticated translocation mechanism, called ‘tethered tracking’, to slide in a 5–3′ direction along nascent RNA, dissociating transcriptional R-loops and/or TECs in the process (Figure 1A). This mechanism invokes net translocation steps of one RNA base per hydrolyzed ATP molecule (10–12) and conforms to the general idea that toroidal helicases directionally translocate nucleic acids within their central channel (5).

Rho is an interesting antibacterial target because it is widespread in bacteria and absent from eukaryotes (13). Bicyclomycin (BCM), a natural Rho inhibitor used in veterinary medicine (14), is mostly effective against Gram-negative bacteria (15). Until now, only one Gram-positive species—*Micrococcus luteus*—has been found to be sensitive to BCM (16). Bacterial resistance to BCM may have several origins. Rho may be dispensable in (as for *Staphylococcus aureus*) (17) or absent from (as for ∼8% of bacterial species) (13) a given bacterium. Alternatively, the bacterium may use a specific resistance mechanism, for instance one conferred by a drug efflux protein (18) or by mutations in Rho that alter BCM binding (13,19). It is also conceivable that the mechanism of action of Rho is different and insensitive to BCM in some bacteria. BCM binds in a subsite of the ATPase pocket of _Ec_Rho where it occludes binding of the nucleophilic water molecule required for ATP hydrolysis (15,20). Thus, Rho factors that would not use ATPase-driven, molecular motor activity to trigger transcription termination would be inherently resistant to BCM.

Rho is considered essential for the growth of *Mycobacterium tuberculosis* (21–23), a species that is nonetheless resistant to BCM (24). A recent report suggests that *M. tuberculosis’* Rho (_Mtb_Rho) has no duplex unwinding (helicase) activity and can dissociate TECs without efficient molecular motor action (25). The authors argue that only ATP binding to _Mtb_Rho is required for its transcription termination function because non-hydrolysable adenosine 5′-(β,γ-imido)triphosphate (AMP-PNP) and ATPγS analogs stimulate the dissociation of artificially stalled TECs by _Mtb_Rho. They also observed that BCM inhibits the ATPase activity of _Mtb_Rho (IC_50_ = 30 μM) but fails to significantly affect transcription termination in the tested concentration range (up to 200 μM of BCM) (25). These unexpected findings suggest that _Mtb_Rho uses a mechanism that is different from that of _Ec_Rho and reminiscent of that used by RNA helicases working as local ‘disruptases’.

To test the possibility that _Mtb_Rho uses a molecular switch (e.g. local ‘disruptase’) mechanism that would make it intrinsically resistant to BCM, we have studied the motor activity of _Mtb_Rho in detail using *in vitro* ATPase, duplex unwinding and transcription termination assays as well as a catalytic _Mtb_Rho mutant and _Mtb_Rho/_Ec_Rho domain fusion chimera. We show that _Mtb_Rho is a [5′→3′]-directional, ATP-dependent RNA helicase which requires a catalytic site that is fully competent for ATP hydrolysis to promote duplex unwinding or transcription termination. Non-hydrolysable ATP analogs do not promote duplex unwinding by _Mtb_Rho nor change dramatically _Mtb_Rho affinity for RNA. Furthermore, both the helicase and transcription termination activities of _Mtb_Rho can be inhibited by BCM, albeit at concentrations that are significantly higher than for _Ec_Rho. Experiments with a domain deletion mutant and the _Mtb_Rho/_Ec_Rho chimera indicate that this low apparent BCM affinity is conferred principally by the C-terminal domain (CTD) of _Mtb_Rho while the N-terminal domain (NTD) specifies which regions of nascent RNA transcripts are utilized by _Mtb_Rho. Overall, our data argue against a scenario whereby _Mtb_Rho would rely on a distinctive, motor-independent mechanism (25) and demonstrate that _Mtb_Rho is an ATP-dependent translocase that is well adapted to the peculiar transcriptome and slow metabolism of *M. tuberculosis*. Our findings also suggest that inhibition of the ATPase-dependent activities of _Mtb_Rho could be a valid strategy in search of new antibiotics targeting *M. tuberculosis*.

## MATERIALS AND METHODS

### Materials

Chemicals and enzymes were purchased from Sigma-Aldrich and New England Biolabs, respectively. BCM was purchased from Santa Cruz Biotechnology. Sigma-saturated RNAP from *E. coli* was obtained from Epicentre Biotechnologies. Nucleoside triphosphates were purchased from GE-Healthcare while ADP, AMP-PNP and ATPγS analogs were from Sigma-Aldrich. Radionucleotides were from PerkinElmer. Synthetic oligonucleotides were obtained from Eurogentec. Polynucleotide fragment (> 300 nt) stocks were prepared as described previously (26) from polynucleotide batches obtained from Santa Cruz Biotechnology and Midland Certified Reagent Company (Midland, TX, USA). Streptavidin-coated magnetic beads were from Ademtech (Pessac, France). DNA templates used in *in vitro* transcription reactions were prepared by standard PCR procedures, as described previously (27). RNA substrates were obtained by *in vitro* transcription of PCR amplicons with T7 RNAP and purified by denaturing polyacrylamide gel electrophoresis (PAGE), as described (26). Plasmids for over-expression of single-point _Mtb_Rho mutants were prepared by Quickchange (Stratagene) mutagenesis of the pET28b-MtbRho plasmid (kindly provided by Dr. Ranjan Sen, Hyderabad, India). Plasmids for over-expression of the Δ_Mtb_Rho deletion mutant (wherein amino acids 76–219 of _Mtb_Rho are replaced by a Val-Pro dipeptide) and the _[Mtb:Ec]_Rho (wherein amino acids 1–308 of _Mtb_Rho are fused to amino acids 131–419 of _Ec_Rho) and _[Ec:Mtb]_Rho (wherein amino acids 1–130 of _Ec_Rho are fused to amino acids 309–602 of _Mtb_Rho) chimeras were obtained by inserting synthetic DNA fragments (obtained by overhang extension PCR using the pET28b-Rho (26) and/or pET28b-MtbRho plasmids as starting templates) between the NdeI and XhoI sites of the pET28b plasmid (Novagen) following standard cloning procedures (see Supplementary Table S1 for details). The sequences of DNA templates and plasmids were verified by capillary DNA sequencing (Genoscreen, France).

### Preparation of proteins

The _Ec_Rho protein was prepared and purified as described previously (26). Wild-type (WT) and mutant _Mtb_Rho factors as well as Mtb:Ec protein chimeras were over-expressed as N-terminal His tag fusions (25) in Rosetta 2(DE3) cells (Merck-Millipore) harboring the appropriate pET28b derivative and were purified following published procedures (28). Briefly, the _Mtb_Rho proteins were affinity selected on His-Pur colbalt resin (Thermo scientific) and were subsequently purified by cation exchange chromatography on a SP sepharose FF column (GE-Healthcare) with a 0.15-1M NaCl gradient and by gel filtration chromatography on a Sephacryl S-300 HR column (GE-Healthcare) equilibrated in GF buffer (150 mM NaCl, 20 mM HEPES, pH 7.5, 2 mM β-mercaptoethanol). Protein preparations were controlled by liquid chromatography high-resolution mass spectrometry using an UltiMate 3000 NanoRSLC system (Dionex, Sunnyvale, CA, USA) connected to a 4-GHz MaXis ultra-high resolution quadrupole-TOF spectrometer (Bruker Daltonics) equipped with an electrospray ion source. All proteins were stored at −20°C as micromolar solutions in 100 mM KCl, 10 mM Tris-HCl, pH 7.9, 0.1 mM EDTA, 0.1 mM DTT and 50% (v/v) glycerol. Concentrations of _Ec_Rho and _Mtb_Rho proteins are expressed in hexamers throughout the manuscript. Purified Ded1 protein was kindly provided by Prof. Eckhard Jankowsky (Case Western Reserve University, Cleveland, USA).

### Preparation of duplex substrates

Duplexes were assembled by mixing 10 pmoles of RNA transcript with 12 pmoles of complementary oligonucleotide (bearing a 6-carboxyfluorescein label at the 5′-end) in annealing buffer (150 mM potassium acetate, 20 mM HEPES, pH 7.5, 1 mM EDTA). Mixtures were heated at 95°C and slowly cooled to 20°C. Duplexes were then purified by native 7% PAGE and stored at −20°C in helicase buffer (20 mM HEPES, pH 7.5, 0.1 mM EDTA, 0.5 mM DTT and 150 mM potassium glutamate) (26).

### NTP hydrolysis assay

The NTP hydrolysis activities of the Rho enzymes were determined with the EnzCheck Phosphate Assay kit (Molecular Probes) as described previously (27,28). Briefly, two quartz cuvettes containing equal volumes of a solution of 20 nM Rho, RNA cofactor (e.g. poly[rC] at a standard concentration of 10μM in rC residues), 0.4 mM 2-amino-6-mercapto-7-methylpurine riboside and 1 U/mL of purine nucleoside phosphorylase in NTPase buffer (1 mM MgCl_2_, 50 mM Tris-HCl, pH 7.5, 0.1mM sodium azide) were placed in the control and sample beam holders of an Uvikon-XL UV-spectrophotometer equipped with a 37°C circulating water bath. After equilibration at 37°C for 5 min, equal volumes of water and NTP (1 mM, final concentration) were added, respectively, to the control and reaction cuvettes and absorbance at 360 nm was recorded as a function of time. The NTPase rates were deduced from the initial first-derivative maxima that were obtained after smoothing of the time-dependent A_360_ curves with Kaleidagraph software. The experiments were calibrated with standard samples containing known amounts of phosphate, as recommended by the kit manufacturer.

### Equilibrium binding assays

Equilibrium Rho-RNA dissociation constants were determined using a filter-binding assay, as described previously (29,30). Briefly, ∼10 fmoles of ^32^P-labeled RNA substrate were mixed with various amounts of Rho in 100 μl of binding buffer (20 mM HEPES, pH 7.5, 0.1 mM EDTA, 0.5 mM DTT, 150 mM potassium acetate and 20 μg/ml BSA) containing 0 or 1 mM ATP (or AMP-PNP). After incubation for 10 min at 30°C, the samples were filtered through stacked [top] nitrocellulose (Amersham Protran) and [bottom] cationic nylon (Pall Biodyne B) membranes using a Bio-dot SF apparatus (Biorad). No significant differences were observed in control experiments performed with 30 min incubation times (not shown). The fractions of free and Rho-bound RNA (retained on the nylon and nitrocellulose membranes, respectively) as a function of Rho concentration were then determined by phosphorimaging of the membranes using a Typhoon-Trio imager and dedicated ImageQuant TL v8.1 software (GE healthcare).

### Duplex unwinding assay

Helicase reactions were performed as described previously (31) with 6-carboxyfluorescein labeled duplex substrates (control experiments with ^32^P-labeled substrates supported that the fluorescein moiety does not affect _Mtb_Rho behavior) (28). Briefly, 5 nM substrate was mixed with 50 or 500 nM Rho and 0–300 μM BCM in helicase buffer and incubated for 3 min at 30°C. Then, 1 mM MgCl_2_, 1 mM nucleotide cofactor (ATP, ADP, ATPγS, AMP-PNP, ADP-BeF_x_ or ADP-AlF_4_) and 400 nM oligo trap (unlabeled oligonucleotide having the same sequence than the released ‘reporter’ strand) were added to the helicase mixture before further incubation at 30°C. Reaction aliquots were taken at various times and mixed with two volumes of quench buffer (10 mM EDTA, 1.5% SDS, 300 mM sodium acetate, 6% Ficoll-400) before being loaded on 9% polyacrylamide gels that contained 1X TBE and 0.5% SDS. Detection and quantification of gel bands were performed by fluorescence imaging with a Typhoon-Trio imager as described (28). Helicase reaction parameters were obtained by fitting data points to an equation describing the kinetic regimen uncovered previously for _Ec_Rho:

\begin{equation*} {F}_{\rm p} = {A} \times (1 - {e}^{ - {k}_{\exp } {t}} ) + {k}_{{\rm lin}} \times {t} \end{equation*}

where *F*_p_ is the fraction of product formed, A is the amplitude of the exponential (burst) phase of the reaction, and *k*_exp_ and *k*_lin_ are the rate constants of the exponential and linear phases of the reaction, respectively (30,32).

### Transcription termination experiments

Transcription termination experiments were performed under single-round ‘chase’ conditions as described previously (33) with minor modifications. Briefly, biotinylated DNA templates were prepared by PCR amplification of pT7A1-λ*cro* DNA template (34) using a 5′-biotinylated reverse primer. To allow preparation of TECs halted at +24, a forward mutagenic PCR primer was also used to introduce a C→T mutation at position +6 of the pT7A1-λ*cro* DNA template. The resulting mAS97Bt template (30 nM) was mixed with *E. coli* RNAP holoenzyme (36 nM) in transcription initiation buffer (40 mM Tris-HCl, pH 7.9, 50 mM KCl, 5 mM MgCl_2_, 1mM DTT, 0.05 mg/ml BSA, 0.4U/μl Superase-In [Ambion]) and incubated at 37°C for 5 min. The mixture was then supplemented with 12 μM ApU, 6 μM ATP, 6μM GTP, 0.95μM UTP, and 1 μCi/μl [α^32^P]-UTP and incubated for 10 min at 37°C. Then, the mixture was chilled on ice, KCl concentration was raised to 0.1 M and the halted transcription complexes were purified by filtration through a microspin G50 column pre-equilibrated with transcription elongation buffer (40 mM Tris-HCl, pH 7.9, 100 mM KCl, 5 mM MgCl_2_, 1 mM DTT, 0.1 mg/ml BSA, 0.2U/μl Superase-In). The complexes were then immobilized on streptavidin beads (Supplementary Figure S4) or used directly in single-run chase reactions. In the latter case, an aliquot of the solution of halted transcription complexes (∼15 nM) was supplemented with 70 nM Rho and 0–750 μM BCM in transcription elongation buffer and incubated for 10 min at 37°C. Then, rNTPs (75 μM each, final concentrations) and rifampicin (25 μg/ml) were added to initiate chase reactions. After incubation for 10 min at 37°C, the mixtures were mixed with 1 volume of denaturing buffer (95% formamide, 50 mM EDTA) and resolved by 8–10% denaturing PAGE. Gels were analyzed by Typhoon-Trio phosphorimaging. Apparent global termination efficiencies (TE_app_) were defined as:

\begin{equation*} {\rm TE}_{{\rm app}} = \frac{{\sum {{I}_{{\rm term}} } }}{{\sum {{I}_{{\rm term}} } + \sum {{I}_{{\rm runoff}} } }} \times 100 \end{equation*}

where ∑ *I*_term_ is the sum of the intensities of the bands corresponding to termination products while ∑ *I*_runoff_ is the sum of the intensities of the bands corresponding to runoff products. IC_50_ values for BCM inhibition were obtained by non-linear least-square fitting of TE_app_ values obtained as a function of BCM concentration to a standard sigmoid inhibition equation:

\begin{equation*} {\rm TE}_{{\rm app}} = ({\rm TE}_{{\rm app}} )_0 - {\rm F}_{\max } \times \frac{{[{\rm BCM}]^{n} }}{{[{\rm BCM}]^{n} + [{\rm IC}_{50} ]^{n} }} \end{equation*}

where (TE_app_)_0_ is the value of TE_app_ at 0 μM BCM, *F*_max_ is the maximal fraction of TE_app_ that is sensitive to BCM inhibition and *n* is an empirical parameter that defines the sigmoid shape of inhibition (35).

### Data reporting

All data values are reported as mean ± standard deviation calculated from at least two independent experimental replicates (whenever number of replicates *N*_R_ > 2 is indicated in figure legends).

## RESULTS

### Selection of protein mutants and chimeras to probe _Mtb_Rho features

The typical _Ec_Rho ring is made of six identical protein subunits, each divided in two distinct domains (7–9,36,37). The NTD contains primary binding site (PBS) motifs involved in the recruitment of RNA *Rut* (Rho utilization) sites by the Rho hexamer (Figure 1A and B). PBS motifs include residues forming a cleft only large enough to accommodate a 5′-YC dimer (Y and C being pyrimidine and cytosine residues, respectively), thereby explaining the preference of _Ec_Rho for single-stranded C-rich *Rut* sites (7–9,37). The CTD contains residues required for intersubunit cohesion and for ‘molecular motor’ function. Essential CTD motifs include Walker A and B motifs forming the ATP binding pockets at subunit interfaces, ‘catalytic Glu’, ‘Arg valve’ and ‘Arg finger’ residues required for the catalysis of ATP hydrolysis as well as Q-loop and R-loop residues forming the secondary binding site (SBS) for RNA (Figure 1B) (10,38). Importantly, changes in the chemical state of the ATPase pockets are allosterically transmitted up to the SBS residues which are in charge of RNA translocation within the central ring channel (Figure 1A) (10).

Alignment of Rho sequences from over 1200 bacterial species indicates that most of these key residues are conserved across the bacterial kingdom, including in phylodistinct Rho factors such as _Mtb_Rho (Supplementary Figure S1) (13). Notwithstanding, _Mtb_Rho displays a few distinctive features that could explain its uncharacteristic behavior. These features include (i) a 144 amino acids (aa) insertion subdomain in the NTD (Figure 1 B) that should result in bulky appendages to the hexameric ring (Figure 1C, in magenta); (ii) a short insert (aa 281–286) between PBS residues (Figure 1B and Supplementary Figure S1) that could affect the structure of the 5′-YC binding clefts (Figure 1C, in red) and recognition of *Rut* sites; and (iii) a threonine instead of a lysine at an SBS position in the R-loop (Thr_501_ in _Mtb_Rho versus Lys_326_ in _Ec_Rho; Supplementary Figure S1 and Figure 1C, in green) that could weaken the allosteric interaction network critical for Rho mechanochemistry.

To probe the effect of these distinctive features, we prepared _Mtb_Rho derivatives containing a single-point SBS mutation (T501A and T501K mutants) or lacking the NTD insertion subdomain (Δ_Mtb_Rho) and a protein chimera wherein the NTD of _Mtb_Rho is fused to the CTD of _Ec_Rho (_[Mtb:Ec]_Rho chimera). Multiple attempts to prepare the ‘reverse’ _[Ec:Mtb]_Rho chimera (NTD of _Ec_Rho fused to the CTD of _Mtb_Rho) were unsuccessful because this protein was invariably expressed in inclusion bodies and could not be purified in a soluble form under native conditions (data not shown).

To probe the role of ATP hydrolysis in _Mtb_Rho, we also prepared a single-point mutant wherein the catalytic glutamate residue Glu_386_ is replaced by alanine (E386A mutant). The role of the catalytic glutamate residue is to polarize the nucleophilic water molecule for attack of the γ-phosphoryl group of ATP (10). The non-conservative mutation of this critical residue in _Ec_Rho (E211A mutation) does not affect ATP binding or protein oligomerization but completely abolishes ATP hydrolysis (38).

### Tight allosteric coupling within the _Mtb_Rho enzyme

**Figure 2.**
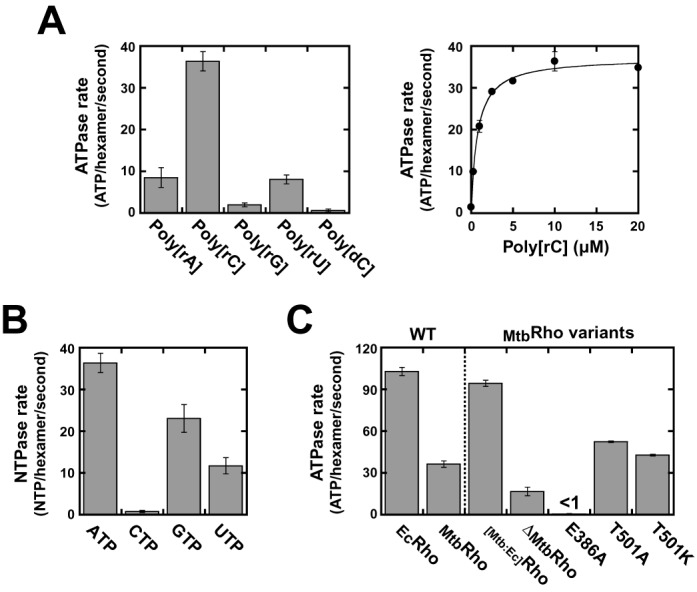
RNA-dependent NTP hydrolysis activities of the _Mtb_Rho protein variants. Unless specified otherwise, the concentrations of NTP and RNA polymer were 1 mM and 10 μM (in nucleotide residues), respectively. (**A**) Stimulation of the ATPase activity of WT _Mtb_Rho by polyribonucleotide cofactors (number of replicates, *N*_R_ = 4 for 10 μM poly[rC]). (**B**) WT _Mtb_Rho hydrolyzes ATP most efficiently in the presence of poly[rC] (*N*_R_ = 4 for ATP). (**C**) Comparison of the ATPase rate of WT _Mtb_Rho with rates measured under the same standard conditions for WT _Ec_Rho and _Mtb_Rho variants (*N*_R_ = 3 for Δ_Mtb_Rho and *N*_R_ = 4 for WT _Mtb_Rho).

To assess the substrate/cofactor preferences of _Mtb_Rho, we performed standard measurements of its NTP hydrolysis rate (27) in the presence of various NTP substrates and polynucleotide cofactors. In agreement with previous reports (25,39), we found that an RNA cofactor is required to activate NTP hydrolysis by _Mtb_Rho (Figure 2A and data not shown). Poly[rC] is the preferred polynucleotide cofactor with a *K*_m_ of ∼0.7 μM (Figure 2A) while ATP is the preferred substrate (Figure 2B). Hydrolysis of CTP is highly inefficient (Figure 2B) at a concentration (1 mM) that is saturating for ATP (*K*_m, ATP_ ∼ 70 μM) (25), a feature that has also been observed for the Rho factors of *M. luteus* (_Mlut_Rho) (40) and *Streptomyces lividans* (41), two evolutionary relatives. By contrast, the phylodivergent _Ec_Rho displays little preference for a specific NTP (40).

Under our standard conditions (see ‘Materials and Methods’ section), the rate of ATPase turnover is about 3-fold lower for _Mtb_Rho than for _Ec_Rho or for the _[Mtb:Ec]_Rho chimera (Figure 2C), suggesting that components in the CTD of _Mtb_Rho are suboptimal. Deletion of the NTD insertion subdomain of _Mtb_Rho (Δ_Mtb_Rho) reduces the ATPase rate by half under standard conditions (Figure 2C). However, a higher concentration of poly[rC] cofactor is required to reach maximal ATPase turnover with the Δ_Mtb_Rho variant (data not shown), which is consistent with an RNA binding defect induced by the deletion of the NTD subdomain (see Table 1 and below). At saturating poly[rC] concentrations, Δ_Mtb_Rho hydrolyzes ATP at a rate of ∼80 molecules per hexamer and per second, which is about twice faster than WT _Mtb_Rho (Figure 2C) and suggests that the NTD subdomain somewhat contributes to regulate ATP consumption.

**Table 1.** RNA binding parameters^a^

Rho factor	No nucleotides		+ AMP-PNP		+ ATP	
	*n*	*K*_d_ (nM)	*n*	*K*_d_ (nM)	*n*	*K*_d_ (nM)
WT _Mtb_Rho	1.2 ± 0.2	3.5 ± 0.7	0.9 ± 0.2	2.0 ± 0.3	1.0 ± 0.2	2.4 ± 0.6
E386A	1.5 ± 0.3	5.3 ± 0.8	0.9 ± 0.2	2.2 ± 0.4	1.1 ± 0.4	3.3 ± 1.0
Δ_Mtb_Rho	2.3 ± 0.6	20.4 ± 2.3	1.4 ± 0.2	16.3 ± 0.4	1.7 ± 0.3	14.4 ± 1.0

^a^Values were determined from the best non-linear least square fits of binding data to the Hill equation, *F* = *F*_∞_ × [Rho]*^n^*/(*K*_d_*^n^* + [Rho]*^n^*), where *F* is the fraction of labeled substrate retained on the nitrocellulose membrane, *F*_∞_ is the maximal fraction of bound substrate, *n* is the Hill coefficient and *K*_d_ is the apparent dissociation constant. Experiments were performed with the *Rut*-containing RNA strand of helicase substrates (Figure 3) and the indicated _Mtb_Rho variants as described in methods.

Altering the identity of Thr_501_ in _Mtb_Rho increases the rate of ATP hydrolysis by 1.2–1.4-fold (Figure 2C, T501A and T501K mutations). This modest effect suggests that Thr_501_ has a less important role in _Mtb_Rho than has the corresponding Lys_326_ side chain in the R-loop of _Ec_Rho (the K326A mutation reduces the ATPase activity of _Ec_Rho by ∼75%) (10,27). In sharp contrast, non-conservative mutation of the catalytic Glu_386_ residue (E386A mutant) completely inhibits ATP hydrolysis by _Mtb_Rho, even when concentrations of ATP and poly[rC] are increased to 5 and 30 μM, respectively (Figure 2C and data not shown). This dramatic effect induced by the E386A mutation is not matched by a drastic change of the oligomeric state or capacity to bind ATP or RNA (Supplementary Figure S2 and Table 1). Thus, the E386A phenotype is comparable to that of the inactivating E211A mutation in _Ec_Rho (38) which supports that the conserved catalytic glutamate residue (Supplementary Figure S1) is also required in _Mtb_Rho for polarization of the water molecule attacking the γ-phosphoryl group of ATP.

Taken together, these data establish that coupling between RNA binding and ATP hydrolysis is tight within the _Mtb_Rho ring and most likely relies on similar principles in the _Mtb_Rho and _Ec_Rho enzymes. One should recall, however, that the rate of ATP hydrolysis is a measure of fuel consumption but by no means a measure of motor efficiency. The latter depends on multiple factors governing the mechanochemical transduction mechanism and yield (42), some of which may vary between the _Mtb_Rho and _Ec_Rho motors.

### _Mtb_Rho unwinds short RNA duplexes in a directional, ATP-dependent fashion

**Figure 3.**
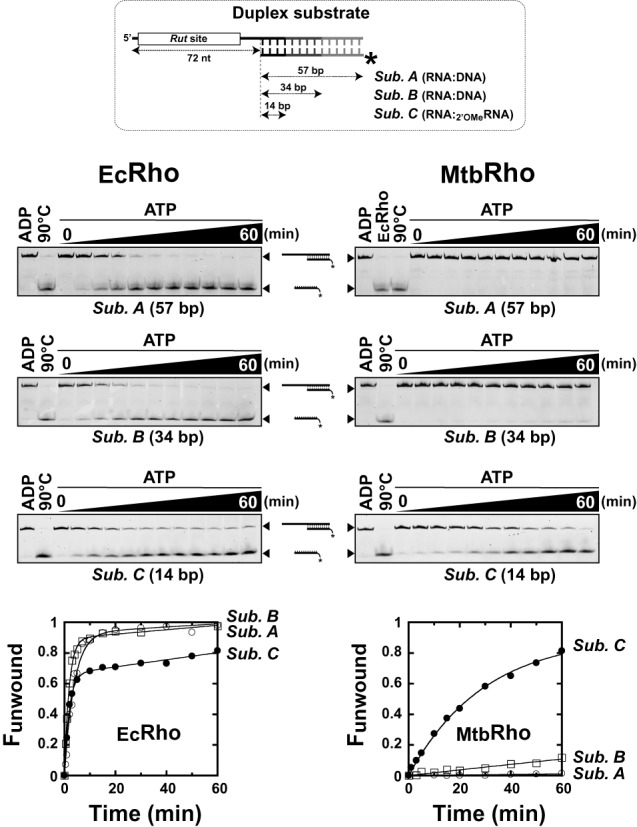
The _Mtb_Rho helicase unwinds short duplex substrates containing a 5′-single-stranded RNA tail. The duplex substrates used in helicase assays contain a synthetic *Rut* site for _Ec_Rho (30) and are schematically depicted at the top of the figure. Unwinding reactions were performed under standard conditions with 50 nM of the _Ec_Rho (left) or _Mtb_Rho (right) helicase and 5 nM of the indicated substrate. Representative 9% PAGE gels and fraction unwound versus time graphs illustrate the helicase activities observed in each case (*N*_R_ = 3 except for experiments with _Mtb_Rho and substrate C [*N*_R_ = 5]).

To probe the motor properties of _Mtb_Rho, we performed duplex unwinding (helicase) experiments with model substrates (26). We prepared RNA:DNA substrates containing a 57 bp (substrate A) or 34 bp (substrate B) duplex region located downstream from a single-stranded RNA (ssRNA) tail encompassing a synthetic *Rut* site (Figure 3, inset) (30). We also prepared a 5′-ssRNA tailed substrate containing a shorter duplex (14 bp, substrate C) by pairing an oligo(2′-O-methylribonucleotide) to the RNA strand (Figure 3, inset). The 2′-O-methyl modifications, which increase RNA duplex stability significantly (43), prevent spontaneous denaturation of substrate C under our experimental conditions (Figure 3, ADP lanes in bottom gels).

We incubated each substrate with a 10-fold molar excess of Rho hexamers in the presence of ATP under ‘standard’ helicase reaction conditions (see ‘Material and Methods’ section) (30,32). As expected, _Ec_Rho unwinds the three duplex substrates with high efficiencies (Figure 3, left gels and graph). Substrate C is slightly less reactive than the longer duplex substrates, probably because its 2′-O-methyl moieties favor an A-form duplex conformation and non-productive interactions with _Ec_Rho (31). By contrast, _Mtb_Rho unwinds substrate C most efficiently, unwinds substrate B very inefficiently, and is completely unable to unwind the 57 bp-long duplex of substrate A (Figure 3, right gels and graph). Incubation of the substrates with larger excesses (100-fold) of _Mtb_Rho does not change unwinding kinetics significantly (data not shown), indicating that _Mtb_Rho was already present at a saturating concentration. These data demonstrate that _Mtb_Rho is a RNA helicase, albeit one that works on a narrower range of substrates than _Ec_Rho.

**Figure 4.**
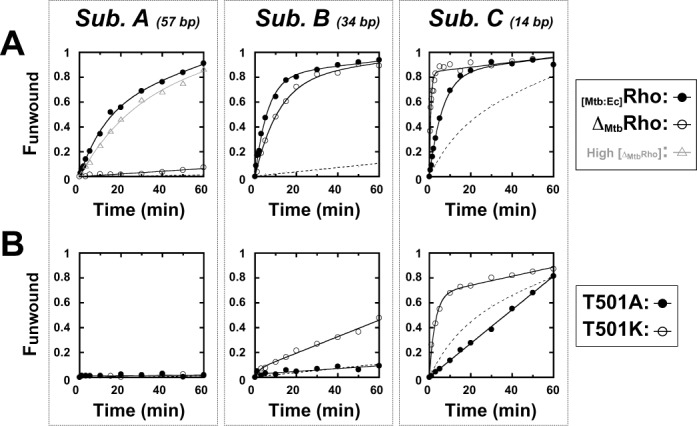
Helicase activities of the _Mtb_Rho protein variants. Unwinding experiments were performed under standard conditions with substrate A, B or C (Figure 3, inset) and (**A**) the _[Mtb:Ec]_Rho chimera and Δ _Mtb_Rho deletion mutant or (**B**) single-point mutants T501A and T501K. To facilitate comparisons, reaction time-courses for WT _Mtb_Rho are depicted as dotted curves.

This narrow spectrum of activity may be due to suboptimal interactions between _Mtb_Rho and the substrates. Optimal *Rut* features for _Mtb_Rho are currently unknown and may not be adequately represented by our synthetic *Rut* sequence (Figure 3, inset), which had been optimized for _Ec_Rho (30,44). Furthermore, the interaction network promoting RNA translocation may be weakened in _Mtb_Rho by the K→T substitution at position 501 of the R-loop (corresponding to SBS residue Lys_326_ in _Ec_Rho; see above). To test these scenarios, we performed unwinding experiments with _Mtb_Rho protein variants. We observed that the _[Mtb:Ec]_Rho chimera unwinds the three substrates with efficiencies that approach the ones displayed by _Ec_Rho (Figure 4A, filled circles; compare with Figure 3). The ATPase rates of _[Mtb:Ec]_Rho and _Ec_Rho are also comparable (Figure 2C). These data indicate that _Mtb_Rho's NTD is reasonably functional (and able to use our synthetic *Rut* sequence) when compared to _Ec_Rho's NTD. Notwithstanding, deletion of the NTD insertion subdomain (Δ_Mtb_Rho variant) also significantly increase the capacity of _Mtb_Rho to unwind the 14 bp and 34 bp duplex substrates (Figure 4A, open circles). When present at a 10-fold higher concentration (500 nM), the Δ_Mtb_Rho variant can even unwind the 57 bp duplex of substrate A with reasonable efficiency (Figure 4A, open triangles) which, as stated above, is not the case of WT _Mtb_Rho. These data combined with equilibrium binding measurements (Table 1) confirm that the NTD insertion subdomain stimulates RNA binding but interferes with motor function.

The T501A and T501K mutations have distinct effects on the helicase activity of _Mtb_Rho. The T501A mutant is slightly less active than the WT enzyme with the shortest (14 bp) duplex substrate and similarly inefficient with the longer duplexes (Figure 4B, filled circles). By contrast, the T501K mutant is significantly more active and able to unwind a longer (34 bp) duplex than WT _Mtb_Rho (Figure 4B, open circles). Moreover, the amplitude of the exponential phase of the unwinding reaction (with Substrate C) is null for T501A but is ∼2-fold higher for T501K than for WT (Figure 4B). It has been shown for _Ec_Rho that the amplitude of the exponential phase depends on helicase processivity under similar unwinding reaction conditions (30,32). These observations suggest that stabilizing, productive contacts are formed between the R-loop and RNA within the SBS of _Mtb_Rho but that these contacts are suboptimal unless Thr_501_ is replaced by a lysine. Significantly, a lysine or arginine is found at the corresponding R-loop position in the Rho factors from most species (e.g. Lys_326_ in _Ec_Rho) (13), with the notable exception of mycobacteria and coryneform relatives (data not shown). Taken together, these findings indicate that features weakening the _Mtb_Rho helicase (with respect to the _Ec_Rho prototype) are located both in its N-terminal ‘RNA anchoring’ and in its C-terminal ‘motor’ domains.

Selective unwinding of short duplexes by _Mtb_Rho is reminiscent of the activity of DEAD-box proteins which disrupt short RNA duplexes in a local, non-directional fashion. However, _Mtb_Rho is unable to unwind a substrate C derivative wherein the relative positions of the *Rut*-containing ssRNA and 14bp duplex regions are permuted (Supplementary Figure S3). The _Ec_Rho enzyme also has no [3′→5′] unwinding activity (30) whereas DEAD-box protein Ded1 unwinds substrate C and its permuted derivative with similar high efficiencies (Supplementary Figure S3), as expected (45). These data thus support that _Mtb_Rho is a directional [5′→3′] helicase behaving more like the _Ec_Rho translocase than the local ‘disruptase’ Ded1.

### ATP hydrolysis is required for duplex unwinding by _Mtb_Rho

A hallmark of RNA translocases is their tight dependence on NTP hydrolysis. By contrast, local disruptases such as DEAD-box proteins often do not require ATP hydrolysis to unwind short RNA duplexes provided that their RNA clamp conformation is stabilized by ATP or a non-hydrolysable ATP analog (46,47). A similar capacity has been proposed for _Mtb_Rho which is able to disrupt an artificially stalled TEC in the presence of AMP-PNP or ATPγS, albeit at rates that are only, respectively, ∼7 and ∼23% of the dissociation rate measured in the presence of ATP (25). We performed RNA affinity measurements to test this proposal but observed that _Mtb_Rho:RNA complexes are only marginally stabilized by nucleotides such as AMP-PNP or ATP (Table 1).

**Figure 5.**
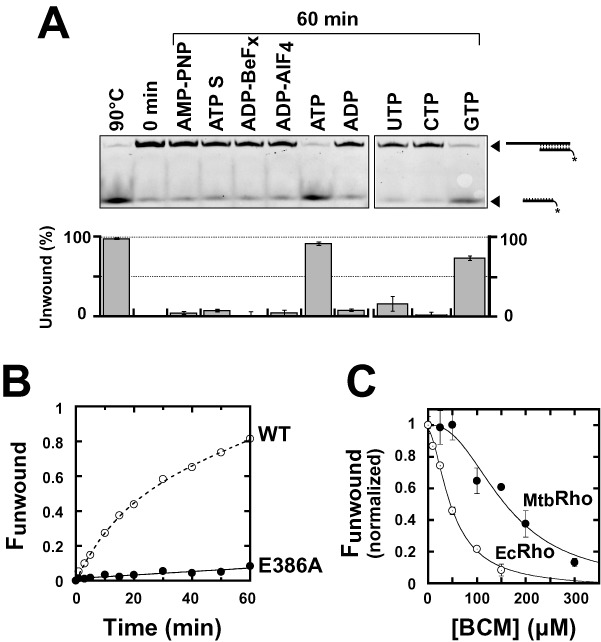
ATP hydrolysis is required for duplex unwinding by _Mtb_Rho. Experiments were performed with substrate C under standard helicase conditions unless specified otherwise. (**A**) Comparison of the activation potential of various nucleotide cofactors (each present at a final concentration of 1 mM). (**B**) The ATPase-deficient mutant E386A does not unwind substrate C efficiently. (**C**) BCM-induced inhibition of the _Ec_Rho (open circles) and _Mtb_Rho (filled circles) helicases. The graph shows the normalized fractions of unwound duplex C formed after 60 min of incubation in the presence of Rho and ATP as a function of the concentration of BCM present in the reaction mixture. Note that BCM has complex effects on the kinetic regimens of the multirun helicase reactions (not shown) which prevent determination of reliable inhibitory parameters based on the evolution of unwinding reaction amplitudes or rates as a function of BCM concentration.

To explore the relationship between the ATPase activity and motor function of _Mtb_Rho, we also carried out unwinding experiments with the 14 bp duplex substrate C (Figure 3) in the presence of various analogs of ATP. We found that non-hydrolysable ATP analogs AMP-PNP, ADP-BeF_x_ (ground-state mimic), and ADP-AlF_4_ (transition-state mimic) do not promote unwinding of substrate C by _Mtb_Rho (Figure 5A). Similarly, we observed that the ATPγS analog, which is hydrolyzed by _Mtb_Rho at a ∼15-fold lower rate than ATP under our standard conditions (data not shown), does not stimulate the _Mtb_Rho helicase significantly (Figure 5A). UTP and CTP are also highly inefficient helicase cofactors whereas GTP is only slightly less efficient than ATP at promoting unwinding of substrate C by _Mtb_Rho (Figure 5A). Overall, the order of NTP efficiency is very similar for duplex unwinding (Figure 5A) and for NTP hydrolysis by _Mtb_Rho (Figure 2B).

The catalytic mutant E386A does not unwind substrate C significantly (Figure 5B) which confirms that ATP hydrolysis is required for the helicase function of _Mtb_Rho. Further support for this conclusion comes from the observation that BCM inhibits the _Mtb_Rho helicase in a dose-dependent fashion (Figure 5C), albeit with a half-maximal inhibitory concentration (IC_50_ = [157 ± 20] μM) that is higher than for _Ec_Rho (IC_50_ = [49 ± 5] μM). Altogether, these data support that _Mtb_Rho is a *bona fide* ATP-dependent molecular motor which requires ATP hydrolysis to trigger duplex unwinding.

### Transcription termination requires a catalytically-competent ATPase pocket in _Mtb_Rho

**Figure 6.**
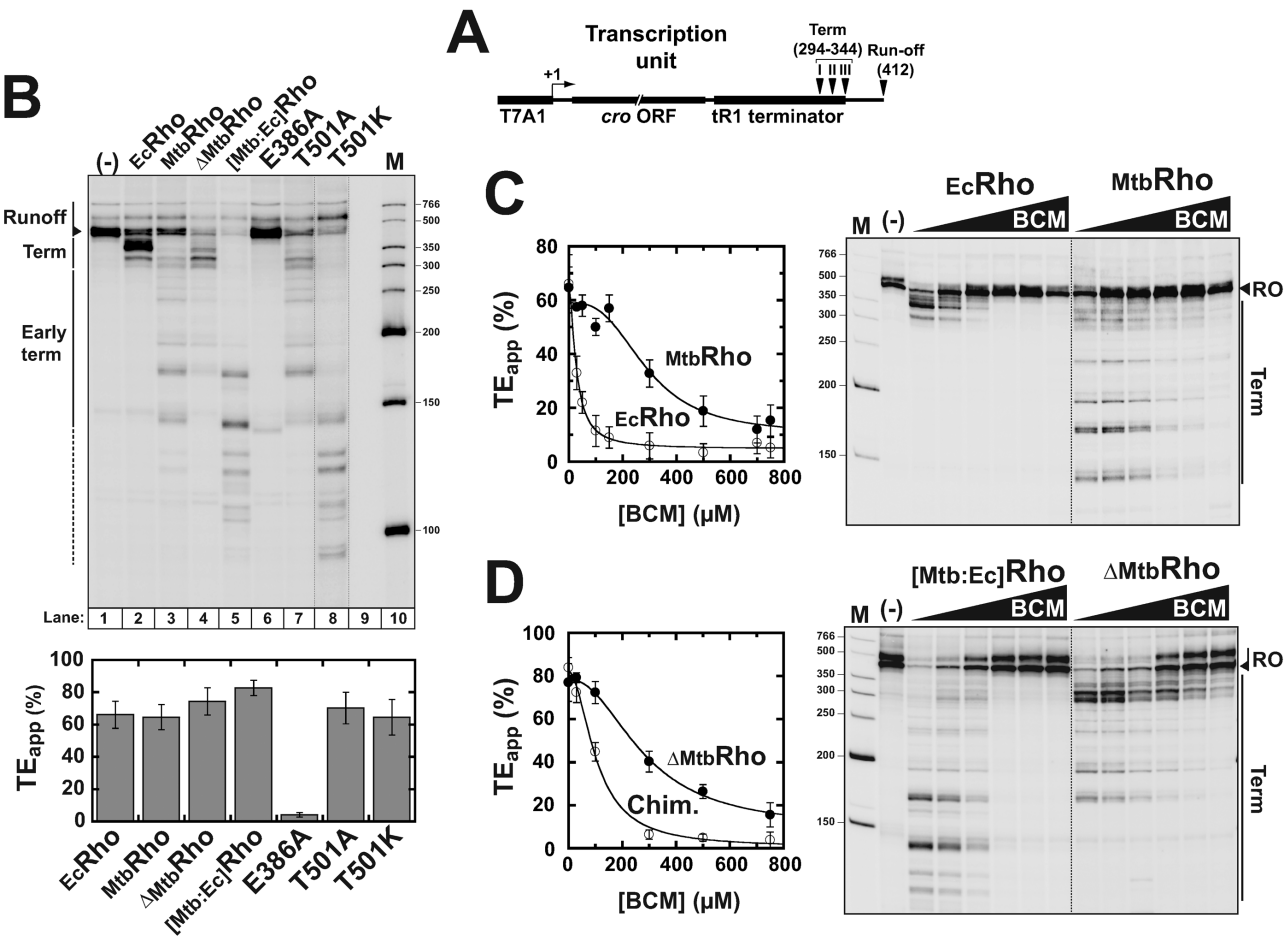
Transcription termination experiments. (**A**) Schematic of the DNA template containing the λtR1 terminator used in *in vitro* termination experiments. (**B**) Both the NTD and CTD of _Mtb_Rho affect its transcription termination activity. A representative denaturing PAGE gel showing the products of transcription termination experiments performed under standard ‘chase’ conditions with *E. coli* RNAP and the λtR1terminator in the presence of various Rho factors (indicated above gel lanes). Intensity of the T501K lane had to be adjusted separately (as indicated by vertical dotted lines) because reactions with the T501K mutant consistently yielded lower product amounts. Apparent termination efficiencies (TE_app_) are indicated below the gel (*N*_R_ = 3). (**C**) A higher concentration of BCM is required to inhibit _Mtb_Rho than _Ec_Rho under standard transcription termination ‘chase’ conditions (*N*_R_ = 3). (**D**) Comparison of the termination activities of the _[Mtb:Ec]_Rho and _ΔMtb_Rho variants in the presence of BCM indicates that structural components conferring BCM resistance to _Mtb_Rho are located mostly in its CTD. In the figure, the intensity and gamma settings were adjusted to facilitate the comparison of termination signals in the various gel panels (separated by dotted lines). Gel images without gamma setting adjustments are presented in Supplementary Figures S6 and S7F.

To assess how the peculiar features of _Mtb_Rho affect its transcription termination activity, we performed *in vitro* transcription termination experiments with the RNAP of *E. coli* and a DNA template encoding the Rho-dependent terminator λtR1 (Figure 6A) (27,34). With this heterologous model system and under single-round ‘chase’ conditions (see ‘Material and Methods’), _Mtb_Rho triggers transcription termination with a global apparent efficiency (TE_app_) that is comparable to that of _Ec_Rho (Figure 6B). However, the termination window (i.e. the region encompassing all RNA release sites) is large and starts much closer to the promoter than for _Ec_Rho (Figure 6B, compare gel lanes 2 and 3; see also Supplementary Figure S4A) which is consistent with previous observations (25).

Promoter-proximal (early) termination stop points are reminiscent of the behavior of the _Mlut_Rho factor from *M. luteus* which also contains a large hydrophilic subdomain in its NTD (Supplementary Figure S1) (40). Deletion of this subdomain in _Mlut_Rho impairs early termination (48). We thus tested the termination activity of the Δ_Mtb_Rho variant, which lacks the NTD insertion subdomain of _Mtb_Rho, and observed that Δ_Mtb_Rho has a reduced capacity to trigger early termination (Figure 6B, lane 4; Supplementary Figures S4A and S5). By contrast, the _[Mtb:Ec]_Rho chimera triggers strong early termination starting even earlier than WT _Mtb_Rho (Figure 6B, lane 5; Supplementary Figures S4 and S5).

Importantly, the catalytic mutant E386A is unable to trigger efficient transcript release (Figure 6B, lane 6; Supplementary Figure S4A) supporting that ATP hydrolysis is mandatory for transcription termination induced by _Mtb_Rho. By contrast, the T501A mutation only marginally affects transcription termination under our standard conditions by slightly favoring late over early termination (Figure 6B, lane 7; Supplementary Figures S4A and S5). Finally, the T501K mutant has an increased capacity to trigger early termination but its TE_app_ is not much different from that of WT _Mtb_Rho under our standard experimental conditions (Figure 6B, lane 8 and histogram; see also Supplementary Figures S4A and S5).

To confirm the importance of ATP hydrolysis for the termination activity of _Mtb_Rho, we performed transcription termination experiments in the presence of increasing concentrations of BCM. We observed that BCM inhibits transcription termination induced by _Mtb_Rho in a dose-dependent fashion (Figure 6C; see also Supplementary Figure S4B) but with a global IC_50_ = [289 ± 51] μM that is one order of magnitude higher than for _Ec_Rho (IC_50_ = [27 ± 3] μM). Quantification of termination efficiencies at individual termination positions yielded similar results with individual IC_50_ values ranging between ∼150 and ∼300 μM for _Mtb_Rho and being lower than ∼40 μM for _Ec_Rho (data not shown). Inhibition of the termination activity of the Δ_Mtb_Rho mutant also requires high BCM concentrations (global IC_50_ = [281 ± 29] μM; Figure 6D), ruling out that BCM activity is perturbed significantly by the NTD insertion subdomain in _Mtb_Rho. By contrast, the _[Mtb:Ec]_Rho chimera is inhibited at significantly lower BCM concentrations with a global IC_50_ of [95 ± 13] μM (Figure 6D) and IC_50_ values for individual termination positions as low as ∼40 μM (Supplementary Figure S6). Taken together, these data indicate that _Mtb_Rho has a low apparent affinity for BCM that is due primarily to the composition of its CTD but confirm nonetheless that _Mtb_Rho requires the energy derived from ATP hydrolysis to trigger transcription termination.

Interestingly, the proportion of late (longer than ∼220 nt) termination products formed with the _[Mtb:Ec]_Rho chimera tend to increase at low BCM concentrations which contrasts with the evolution of early termination products (Figure 6D and Supplementary Figure S6). This suggests that sub-inhibitory concentrations of BCM trigger a downstream shift of the termination window, possibly because BCM slows down processive translocation of the _[Mtb:Ec]_Rho chimera and alters its kinetic competition with RNAP (49). This phenomenon is not detected for WT _Mtb_Rho (Figure 6C and data not shown), which seems consistent with its low apparent processivity deduced from helicase experiments (compare Figures 3 and 4A). Hence, BCM-impaired _Mtb_Rho may not remain bound to the nascent transcript for sufficiently long times (and translocated distances) to catch up with RNAP at alternative pausing sites located further away from the initial *Rut* loading site.

## DISCUSSION

### The mtbRho factor is a *bona fide* molecular motor

Transcription termination factor Rho is widespread in bacteria (13) where it supports important physiological functions linked to Rho-induced disruption of TECs or R-loops (7–9). Many Gram-negative species are sensitive to the antibiotic BCM (9), a naturally occurring, non-competitive inhibitor of Rho's ATPase (20), whereas only one tested Gram-positive species—*M. luteus*—has been found to be sensitive to BCM (16). This spectrum of BCM activity suggested that Rho is dispensable in most Gram-positive bacteria, a proposal that has been tested and verified for only a few specimens such as *S. aureus* (17). Recently, an alternative explanation has been proposed for mycobacterial resistance to BCM (25). Based on the observation that _Mtb_Rho disrupted stalled TECs in the presence of AMP-PNP or ATPγS and because the authors failed to detect a significant helicase activity for _Mtb_Rho, they proposed that the enzyme is able to trigger transcription termination by a mechanism uncoupled from molecular motor function (25). Such a mechanism would make _Mtb_Rho intrinsically resistant to BCM, which is what the authors observed for BCM concentrations up to 200 μM (25). Our data, however, do not corroborate this hypothesis and suggest alternative explanations as is discussed below.

Tight RNA binding by a protein is sometimes sufficient to destabilize proximal complexes on RNA. This principle is utilized by DEAD-box proteins which bind ATP and RNA cooperatively to disrupt short RNA duplexes (or RNA:protein complexes) in a non-directional fashion (1,6). However, _Mtb_Rho does not bind RNA much tighter in the presence of nucleotides (Table 1) and differs from DEAD-box proteins by two other important criteria. First, _Mtb_Rho is unable to unwind short duplex substrates in the presence of non-hydrolysable ATP analogs (Figure 5A). Second, _Mtb_Rho cannot unwind substrates wherein the duplex region is located upstream of the ssRNA loading (e.g. *Rut*) site (Supplementary Figure S3). Rather, the _Mtb_Rho enzyme behaves as a *bona fide* ATP-dependent [5′→3] RNA translocase only able to unwind short RNA duplexes located downstream from a *Rut* site (Figure 3). This activity strictly requires ATP (or GTP) hydrolysis (Figure 5B), a condition confirmed by the inhibition of duplex unwinding induced by the non-conservative mutation of the catalytic Glu_386_ residue (Figure 5B, E386A mutation) or by high concentrations of BCM (Figure 5C). Importantly, *in vitro* transcription termination by _Mtb_Rho is similarly inhibited by the E386A mutation (Figure 6B) or by high BCM concentrations (Figure 6C), supporting that both the helicase and termination functions of _Mtb_Rho proceed by the same motor-dependent mechanism. This conclusion is in apparent contradiction with the previous observation that artificially stalled TECs can be dissociated by _Mtb_Rho in the presence of ATPγS or AMP-PNP (25). However, the reported TEC dissociation rates were low, especially in the presence of AMP-PNP (∼7% of the ATP-dependent rate) (25), and may thus reflect residual motor-independent destabilization as much as experimental limitation(s) due, for instance, to slow hydrolysis of ATPγS by _Mtb_Rho (see ‘Results’ section) or contamination of commercial AMP-PNP preparations by ATP (50).

### Limited helicase processivity versus high termination efficiency of mtbRho

Although our data unambiguously demonstrate that _Mtb_Rho is a directional, ATP-fueled RNA helicase, they also show that the enzyme is much less efficient than its _Ec_Rho homolog at unwinding long duplexes (Figure 3). This low processivity of _Mtb_Rho derives from at least two specific structural features which are discussed below.

First, the presence of a threonine residue (instead of a basic lysine/arginine residue, as in most Rho homologs) (13) at the C-terminal position of the R-loop (Thr_501_) most likely weakens the SBS:RNA interaction network in _Mtb_Rho. Replacement of Thr_501_ by a lysine (T501K mutant) enhances the capacity of _Mtb_Rho to unwind longer duplexes whereas its replacement by alanine (T501A mutant) abrogates the unwinding burst observed with a short duplex (Figure 4B). These observations are consistent with Rho helicase processivity depending on optimal SBS:RNA interactions (11). Interestingly, the T501K mutation also strongly favors early termination whereas T501A slightly disfavors it (Figure 6B; Supplementary Figure S5). The initial capture of a *Rut* site, which depends on PBS:RNA contacts (7–9), should not be affected by modifications of SBS residues. However, subsequent activation of the *Rut*-bound Rho motor may be stimulated by extra (or stronger) SBS:RNA contacts, thereby allowing earlier catch up with RNAP. The superior behavior of the _[Mtb:Ec]_Rho chimera (Figures 4A and 6B), which contains the SBS of _Ec_Rho, lends support to this proposal. One should also note that the suboptimal R-loop configuration in _Mtb_Rho probably increases the dependency of the SBS:RNA network on Q-loop contacts. This conjecture is supported by preliminary analysis of Q-loop mutant phenotypes (our unpublished observations). For instance, the single-point S461A mutant of _Mtb_Rho displays a strong (∼10-fold) defect in poly[rC]-dependent ATPase activity (Supplementary Figure S7) whereas the corresponding T286A mutation in the Q-loop of _Ec_Rho only reduces ATPase activity by 35% (51). This difference should be considered in light of the fact that Thr_286_ side chains make direct contacts with RNA in the crystal structure of _Ec_Rho (10).

Second, the presence of a large, hydrophilic NTD insertion subdomain in _Mtb_Rho (Figure 1B) appears to perturb the coupling between RNA binding and motor functions. Deletion of this subdomain (Δ_Mtb_Rho variant) weakens RNA binding (Table 1) but significantly increases the ATPase turnover and apparent duplex unwinding processivity of _Mtb_Rho at saturating RNA concentrations (see ‘Results’ section and Figure 4A). Bulky NTD subdomain appendages (Figure 1C) may hamper the structural rearrangements driving enzyme activation and mechanochemical transduction (i.e. transformation of chemical energy into mechanical work) in the Rho ring (10,52). Such constraints are, however, much less detrimental in the _[Mtb:Ec]_Rho chimera which displays processive unwinding (Figure 4A) and strong termination (Figure 6B) activities. The NTD subdomain thus appears to specifically exacerbate the suboptimal behavior of the _Mtb_Rho motor.

We cannot totally exclude that the processivity of the _Mtb_Rho helicase is also mitigated by suboptimal PBS interactions with the duplex substrates. Our synthetic *Rut* sequence has been optimized for _Ec_Rho (30,44) and may thus not adequately recapitulate the distinct features of *M. tuberculosis* transcripts (39). An optimal _Mtb_Rho:*Rut* interaction crowning the Rho hexamer could enhance intersubunit cohesion (and ring stability) during tethered tracking, thereby favoring further RNA translocation over RNA exit from the Rho ring and dissociation (11,12). Comparison of the enzymatic activities of the _Mtb_Rho and _[Mtb:Ec]_Rho factors (Figures 2C, 4A and 6) nonetheless supports that _Ec_Rho contains a stronger, more processive motor than _Mtb_Rho. It thus remains to be seen whether _Mtb_Rho will be able to disrupt stable R-loop structures under appropriate conditions. This capacity could have some value if _Mtb_Rho has to deal with transcriptional R-loops *in vivo*, as is the case for _Ec_Rho (Figure 1A) (53), which is not known yet.

Although _Mtb_Rho efficiently terminates transcription by *E. coli*'s RNAP under conditions that favor fast RNAP translocation (Figure 6A; Supplementary Figure S4), our data suggest that _Mtb_Rho cannot operate far from its loading site on the nascent transcript. This proposal is notably supported by the lack of downstream shift of the _Mtb_Rho termination window in the presence of sub-inhibitory concentrations of BCM (see ‘Results’ section). We thus propose that, while the processive _Ec_Rho has evolved to catch up with RNAP at sites located up to ∼130 nt downstream from the *Rut* region (7–9), _Mtb_Rho needs to promptly inactivate its RNAP target. This task could be facilitated by the significantly lower elongation rate of *M. tuberculosis*’ RNAP (54) or by direct interactions between Rho and RNAP (55) that could be stronger in the case of _Mtb_Rho (e.g. due to the NTD insertion subdomain). Further work is necessary to rigorously test these intriguing possibilities.

### Origin(s) of BCM resistance

The low apparent affinity of _Mtb_Rho for BCM that was deduced from helicase (Figure 5C) and transcription termination (Figure 6B and C) experiments is intriguing given that _Mtb_Rho does not contain mutations that are known to confer BCM resistance (13). These mutations usually disrupt molecular contacts between amino acids and BCM in the ATPase pocket of Rho, thereby destabilizing the Rho:BCM complex (20). We could not determine BCM affinities by microcalorimetry because of experimental constraints associated with weak ligand binding (56) and foaming of concentrated _Mtb_Rho solutions in the microcalorimeter mixing chamber (our unpublished observations). Thus, weakening of BCM binding by unknown structural determinants in _Mtb_Rho (e.g. ones that would alter the shape of, or access to, the ATPase pocket) cannot be formally ruled out. Other explanations based on distinctive mechanochemical coupling in _Mtb_Rho are also possible, although their rigorous testing is beyond the scope of the present study.

### Conserved transcription termination mechanisms in *M. tuberculosis*

Pathogenic mycobacteria have long adapted their metabolisms and genetic programs to the colonization of harsh and changing environments such as host macrophages and granuloma (57). How this adaptation is enforced at the level of mycobacterial transcription is only fragmentarily understood. The two major pathways leading to transcription termination in bacteria—that is, intrinsic termination and Rho-dependent termination—have been proposed to be markedly distinct in *M. tuberculosis* (25,58). However, a recent study demonstrated that mycobacterial RNAP terminates transcription at canonical intrinsic terminators in a process which can be aided by NusG, as in other bacteria (54). Mycobacterial RNAP only displayed an increased capacity (as compared to *E. coli*'s RNAP) to use terminators with suboptimal U-tracts (54). In the present work, we demonstrate that _Mtub_Rho operates by a canonical ‘molecular motor’ mechanism that resembles the one used by _Ec_Rho. It thus appears that both intrinsic and factor-dependent termination mechanisms are not fundamentally different in *M. tuberculosis* but adjusted to the characteristics of the bacterium that include a GC-rich genome and slow metabolism. Since _Mtub_Rho is expressed in *M. tuberculosis* (23) and appears to be essential for growth (21,22), seeking _Mtub_Rho inhibitors by using a screening strategy derived from ATP-dependent motor assays (such as the ones presented in this study) could thus prove useful in the fight against tuberculosis, a major and global health concern.

## SUPPLEMENTARY DATA

Supplementary Data are available at NAR Online.

SUPPLEMENTARY DATA
