# Supplementary material for: ATP-dependent motor activity of the transcription termination factor Rho from Mycobacterium tuberculosis
Source: Nucleic Acids Res. 2015 May 20;43(12):6099–111. doi: 10.1093/nar/gkv505 (PMC4499133; doi:10.1093/nar/gkv505)
Supplement: SUPPLEMENTARY DATA [file supp_43_12_6099__index.html]

ATP-dependent motor activity of the transcription termination factor Rho from Mycobacterium tuberculosis — ATP-dependent motor activity of the transcription termination factor Rho from Mycobacterium tuberculosis — SUPPLEMENTARY DATA 

# ATP-dependent motor activity of the transcription termination factor Rho from *Mycobacterium tuberculosis*

## SUPPLEMENTARY DATA

- SUPPLEMENTARY DATA
