# Supplementary material for: ATP-dependent motor activity of the transcription termination factor Rho from Mycobacterium tuberculosis
Source: Nucleic Acids Res. 2015 May 20;43(12):6099–111. doi: 10.1093/nar/gkv505 (PMC4499133; doi:10.1093/nar/gkv505)
Supplement: SUPPLEMENTARY DATA [file supp_gkv505_nar-00884-v-2015-File008.pdf]

ATP-dependent motor activity of the transcription termination factor Rho from *Mycobacterium tuberculosis*

François D'Heygère, Annie Schwartz, Franck Coste, Bertrand Castaing, and Marc Boudvillain

(Supplementary information)

Address correspondence to M.B. (marc.boudvillain@cnrs-orleans.fr)

SUPPLEMENTARY METHODS

Circular Dichroism. CD spectra were recorded at 20 °C on a Jasco J-810 spectropolarimeter from 260 to 195 nm using a 1-mm quartz cell, a scan rate of 50 nm/min, a response time of 8 ms, a bandwidth of 1 nm, a resolution of 1 nm, and a protein concentration of 0.1 mg/ml in a buffer containing 10 mM sodium phosphate, pH 7.5 and 100 mM NaCl. Each spectrum represents the average of five scans, with the buffer subtracted.

Diffusion light scattering. Hydrodynamic radius measurements were made at 20 °C with a Zetasizer Nano S instrument (Malvern Instruments Ltd, UK) and a protein concentration of 1mg/ml in GF buffer.

Supplementary Table 1: primers used for plasmid constructs

Plasmid	Method	Source DNA	Forward primer	Reverse primer
pET28b-MtbRho/T501A	Quickchange [a]	pET28b-MtbRho	5'-GTCGAGACCGGGTCCGCTGGTGACACGGTC'	5'-GACCGTGTCACCAGCGGACCCGGTCTCGAC
pET28b-MtbRho/T501K	Quickchange [a]	pET28b-MtbRho	5'-GGTCGAGACCGGGTCCAAAGGTGACACGGTCATTTTC	5'-GAAAATGACCGTGTCACCTTTGGACCCGGTCTCGACC
pET28b-MtbRho/E386A	Quickchange [a]	pET28b-MtbRho	5'-GTGCTCGTCGACGCGCGGCCTGAGGAG	5'-CTCCTCAGGCCGCGCGTCGACGAGCAC
pET28b-MtbRho/S461A	Quickchange [a]	pET28b-MtbRho	5'-GGCCGGATCCTGGCCGGTGGTGTCG	5'CGACACCACCGGCCAGGATCCGGCC
pET28b-ΔMtbRho	1 [1-75] [b]	pET28b-MtbRho	5'-GGGAATTGTGAGCGGATAAC	5'-CCGGCTGGACGACGTCGTCGGGTACCTGTCGCCTGATCTCC
	2 [220-602] [b]	pET28b-MtbRho	5'-GGAGATCAGGCGACAGGTACCCGACGACGTCGTCCAGCCGG	5'-GGCCCCAAGGGGTTATGCTAGTTATTGCTCAGCGGTGGC
pET28b-[Mtb:Ec]Rho	1 [1-308]] [b]	pET28b-MtbRho	5'-GGGAATTGTGAGCGGATAAC	5'-GGGTTAAGTTCTCAAAGAGGATCCGCTTCTTGGCGTCTTCGACCG
	2 [131-419] [b]	pET28b-EcRho	5'-CGGTCGAAGACGCCAAGAAGCGGATCCTCTTTGAGAACTTAACCC	5'-GGCCCCAAGGGGTTATGCTAGTTATTGCTCAGCGGTGGC
pET28b-[Ec:Mtb]Rho	1 [1-130]] [b]	pET28b-EcRho	5'-GGGAATTGTGAGCGGATAAC	5'-CGTCAGTTTGCCGAACTCGGGTTTGTTGCGGGCGTTTTCAGG
	2 [309-602] [b]	pET28b-MtbRho	5'-CCTGAAAACGCCCGCAACAAACCCGAGTTCGGCAAACTGACG	5'-GGCCCCAAGGGGTTATGCTAGTTATTGCTCAGCGGTGGC

[a] Site-directed mutagenesis using the Quickchange method (Stratagene) and the indicated mutagenic primers.

[b] Overhang extension PCR and standard cloning. Full insert DNA fragments were obtained by fusing the N-terminal fragment 1 to the C-terminal fragment 2 (corresponding Rho amino acids indicated in brackets) by overhang extension PCR. The insert DNA fragments were cloned into the pET28b vector plasmid after digestion of both vector and inserts with the XbaI and XhoI restriction enzymes.

```
                 1                                                         51
M. tuberculosis  MTDTDLITAG ESTDGKPSDA AATDPPDLNA DEPAGSLATM VLPELRALAN RAGVKGTSGM RKNELIAAIE EIRRQAN--- ---------- ----------
M. luteus        MT-------- ESTE------ -------QTT PTNGGGLASL KLAQLQALAS QLGIAGGSRM RKADLVTAIS DHQRGGSVAD RDAAERAAQA PAAPAAETAP
E. coli          ---------- ---------- ---------- -MNLTELKNT PVSELITLGE NMGLENLARM RKQDIIFAIL KQHAK----- ---------- ----------
B. subtilis      ---------- ---------- --------MK DVSISSLENM KLKELYELAR HYKISYYSKL TKKELIFAIL KANAE----- ---------- ----------
                                                                  NHB subdomain

                 101                                                       151
M. tuberculosis  -------GAP AVDRSAQEHD K--------- ---GDRPPSS EAPATQGEQT PTEQIDSQSQ ---------- --QVRPERRS -ATREAGPS- ----------
M. luteus        AAASSEDAAP AAERPARRRS RRADADTSAP AAAQDGQPQA EAREAQTEQA PRETASDQDR SGGSEARDEG EDRPQSERRS RGRRRAGDDD AQQGQDRRSD
E. coli          ---------- ---------- ---------- ---------- ---------- ---------- ---------- ---------- ---------- ----------
B. subtilis      ---------- ---------- ---------- ---------- ---------- ---------- ---------- ---------- ---------- ----------

                 201                                                       251
M. tuberculosis  ---------- ---------- --------GS GERAGTAADD TDNRQGGQQD AKTEERGTDA GGDQGGDQQA SGG------- ----QQARGD EDGEARQGRR
M. luteus        GAQGEDGADA DRRGDREDRD DNGRENGRGR NGRNGRDRDN GRDRENGREN SRDRENGRDG SREQRGDKSE DGGRGDGGRG DRSRRDDRDD EGGRNRRNRR
E. coli          ---------- ---------- ---------- ---------- ---------- ---------- ---------- ---------- ---------- ----------
B. subtilis      ---------- ---------- ---------- ---------- ---------- ---------- ---------- ---------- ---------- ----------

                                                              3'C 3'C            3'C  5'Y                              5'Y
                 301                                                       351
M. tuberculosis  GRRFRDRRRR GERSGDGAEA ELREDDVVQP VAGILDVL-- DNYAFVRT-- SGYLPGPHDV YVSMNMVRKN GMRRGDAVTG AVRVPKEGEQ PNQ------R
M. luteus        NRNERGRNRR GRGGPEVDET ELTEDDVLQP VAGILDVL-- DNYAFVRT-- SGYLPGPNDV YVSLAMVKKY GLRKGDAVVG PIA-PRDGEK QQHHGGGSNR
E. coli          ---------- ---------- ----SGEDIF GDGVLEILQ- DGFGFLRSAD SSYLAGPDDI YVSLSQIRRF NLRTGDTISG KIRPPKEGE- ----------
B. subtilis      ---------- ---------- ----QEDLLF MEGVLEIIQS EGFGFLRP-- INYSPSSEDI YISASQIRRF DLRNGDKVSG KVRPPKENE- ----------
                                                                                    RBD

                   5'Y                  NTD  CTD
                 401                                                       451
M. tuberculosis  QKFNPLVRLD SINGGSVEDA KKRPEFGKLT PLYPNQRLRL ET---STERL TTRVIDLIMP IGKGQRALIV SPPKAGKTTI LQDIANAITR NNPECHLMVV
M. luteus        QKFNALVKIS SVNGQPAVEH PQRVEFGKLV PLYPQERLRL ET---DPKLI GPRVIDLVSP IGKGQRGLIV SPPKAGKTMI LQSIANAIKT NNPEVHLMMV
E. coli          -RYFALLKVN EVNFDKPENA RNKILFENLT PLHANSRLRM ERGNGSTEDL TARVLDLASP IGRGQRGLIV APPKAGKTML LQNIAQSIAY NHPDCVLMVL
B. subtilis      -RYYGLLHVE AVNGDDPESA KERVHFPALT PLYPDRQMVL ET---KPNFL STRIMDMMAP VGFGQRGLIV APPKAGKTML LKEIANSITA NQPEAELIVL
                                                                                           Walker A

                      Bcm                                                            Bcm    Bcm           RNA
                 501                                                       551
M. tuberculosis  LVDERPEEVT DMQRSVKGEV IASTFDRPPS DHTSVAELAI ERAKRLVEQG KDVVVLLDSI TRLGRAYNNA SPASGRILSG GVDSTALYPP KRFLGAARNI
M. luteus        LVDERPEEVT DMQRSVDGEV IASTFDRPAD DHTTLAELAI ERAKRLVEMG RDVVVLLDSM TRLGRAYNLA APASGRILSG GVDSSALYPP KKFFGAARNI
E. coli          LIDERPEEVT EMQRLVKGEV VASTFDEPAS RHVQVAEMVI ERAKRLVEHK KDVIILLDSI TRLARAYNTV VPASGKVLTG GVDANALHRP KRFFGAARNV
B. subtilis      LVDERPEEVT DIERSVAGDV VSSTFDEVPE NHIKVAELVL ERAMRLVEHK KDVIILMDSI TRLARAYNLV IPPSGRTLSG GIDPAAFHRP KRFFGAARNI
            catalytic Glu  Arg Valve                                           Walker B                Q-loop

                                  RNA          Bcm
                 601                                                       651
M. tuberculosis  EEGGSLTIIA TAMVETGSTG DTVIFEEFKG TGNAELKLDR KIAERRVFPA VDVNPSGTRK DELLLSPDEF AIVHKLRRVL SGLDSHQAID LLMSQLRKTK
M. luteus        ENGGSLTILA TALVETGSRM DEVIFEEFKG TGNMELRLSR HLAERRIFPA VDVNASGTRR EEALLSQEEV KIMWKLRRVL SGLEQQQALD LLTNKIKDTA
E. coli          EEGGSLTIIA TALIDTGSKM DEVIYEEFKG TGNMELHLSR KIAEKRVFPA IDYNRSGTRK EELLTTQEEL QKMWILRKII HPMGEIDAME FLINKLAMTK
B. subtilis      EEGGSLTILA TALVDTGSRM DDVIYEEFKG TGNMELHLDR SLAERRIFPA IDIRRSGTRK EELLVPKEHL DRLWSIRKTM S--DSPDFAE KFMRKMKKTK
                              R-loop                                          Arg
                                                                             finger

                 701
M. tuberculosis  NNYEFLVQVS KTTPGSMDSD ---
M. luteus        SNAEFLMLVS KTTLGSKGDD ---
E. coli          TNDDFFEMMK RS-------- ---
B. subtilis      TNQDFFDILN QEWKQANLSS ARR
```

Supplementary figure 1: Alignment of representative Rho sequences (excerpt from Ref. 13). Color coding of major motifs is the same as in Figure 1. Key conserved residues are highlighted in grey. The small PBS insert (aa 281-286) and T501 residue (in R-loop) in $_{Mtb}$Rho are highlighted in light red. Numbers above sequences correspond to positions in the alignment (not in individual sequences).

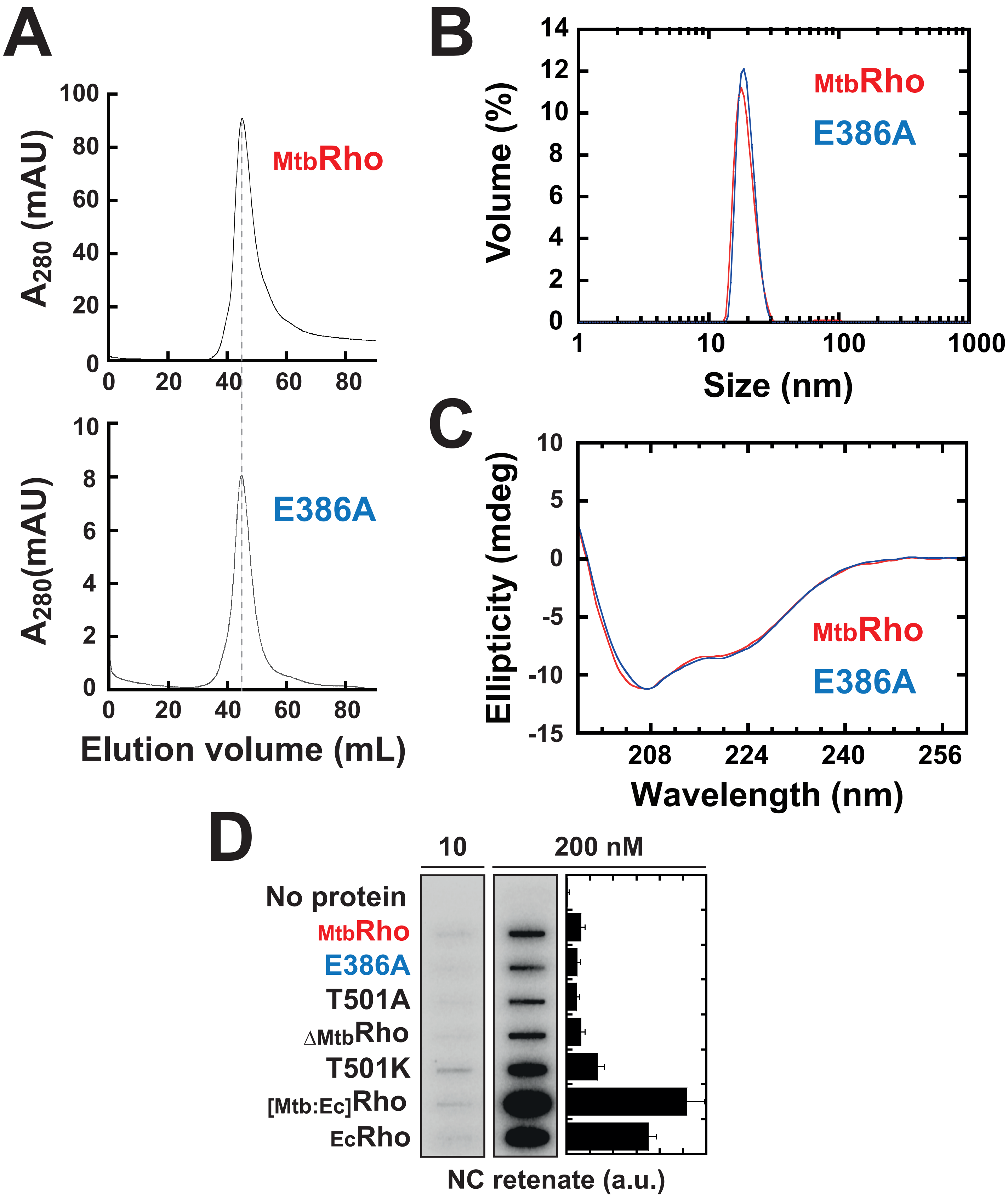

Supplementary figure 2: Single-point mutant E386A and WT $_{Mtb}$Rho have comparable structural, oligomerization, and ATP binding behaviors. **(A)** Chromatograms of the E386A and WT $_{Mtb}$Rho proteins analyzed by gel filtration on a Sephacryl S-300 HR column. **(B)** Diffusion light scattering and **(C)** circular dichroism spectra of the E386A and WT $_{Mtb}$Rho proteins. **(D)** Retention of [γ^{32}P]ATP:Rho complexes on a nitrocellulose (NC) membrane. Rho proteins (10 or 200 nM) were incubated with 0.1 nM [γ^{32}P]ATP in helicase buffer supplemented with 1 mM $MgCl_2$ for 10 min at 30°C before being filtered through a NC membrane using a slot-blot apparatus. Note that the amounts of ATP retained with proteins on the NC membrane are significantly higher for $_{Ec}$Rho and $_{[Mtb:Ec]}$Rho. This suggests that the $_{Ec}$Rho motor binds ATP much tighter than the $_{Mtb}$Rho motor and is consistent with the ~10-fold lower $K_{m,ATP}$ measured for ATP hydrolysis by $_{Ec}$Rho than by $_{Mtb}$Rho (see main text).

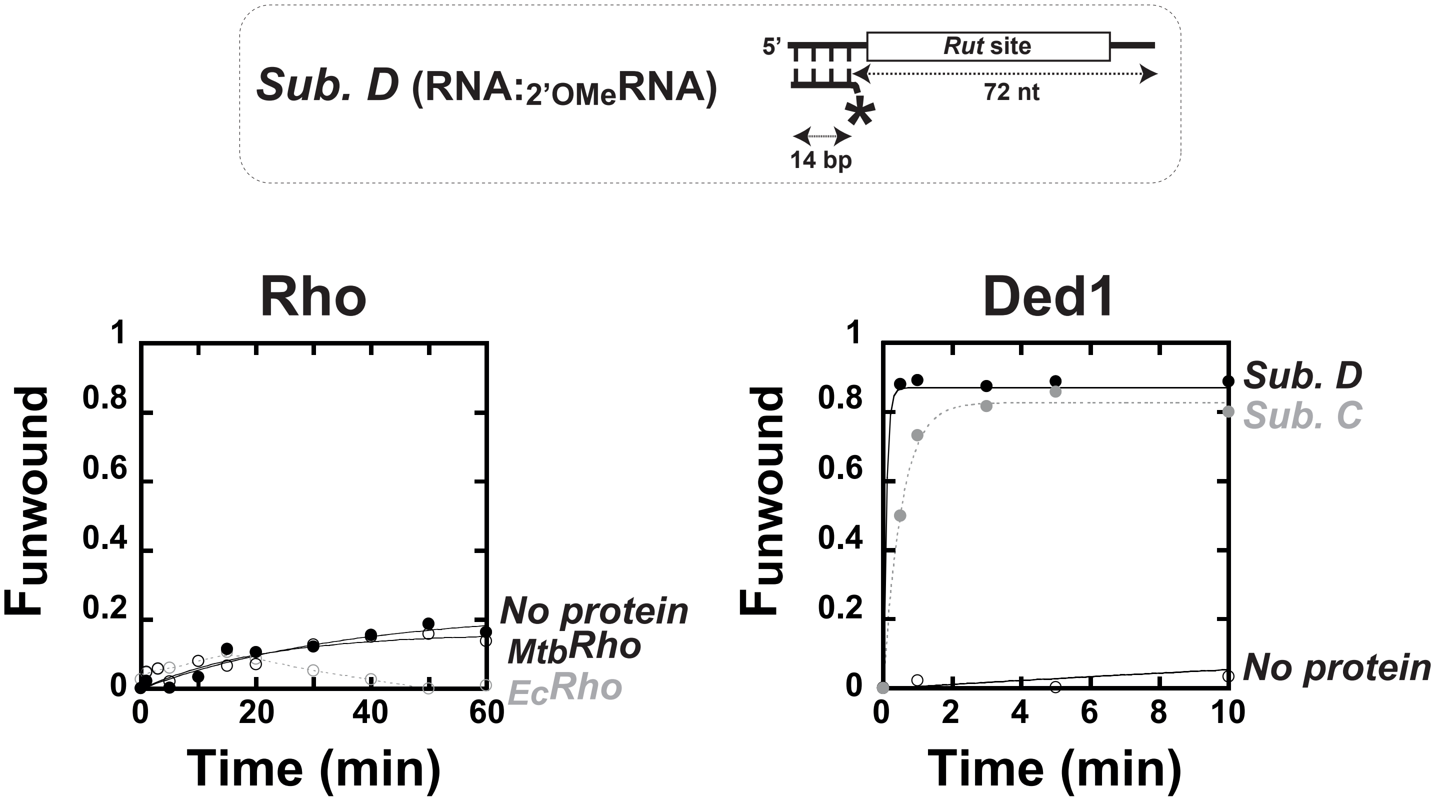

Supplementary figure 3: The $_{Mtb}$Rho enzyme is a directional helicase requiring a 5'-ssRNA tail upstream from its duplex target. Unwinding experiments were performed in the presence of ATP under standard conditions (see methods) with substrate D which contains the same 14bp duplex region than substrate C but located upstream rather than downstream from the ssRNA tail (see diagram, inset). Although the duplex in substrate D is slightly less stable than in substrate C, $_{Mtb}$Rho does not accelerate duplex unwinding with this substrate (left graph). The same is true for $_{Ec}$Rho which even displays a slight 'annealing' activity with substrate D as evidenced by a bell-shaped unwinding curve. By contrast, the non-directional DEAD-box protein Ded1 indiscriminately increases unwinding of the duplexes in substrates C and D. Experiments with Ded1 were performed as described in Ref. 44.

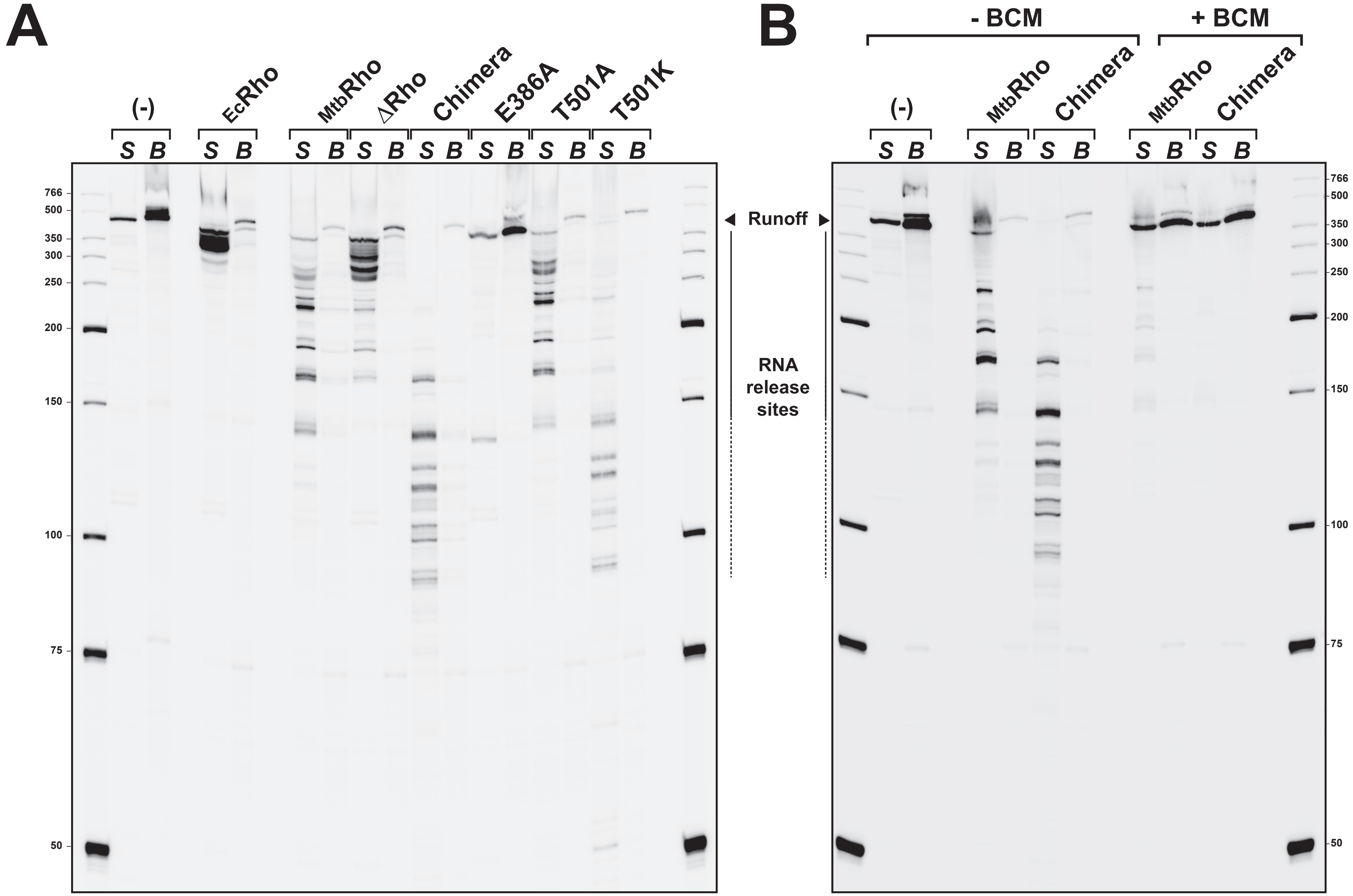

Supplementary figure 4: Single-round transcriptions with bead-affixed transcription complexes containing biotinylated λtR1 DNA templates. The ^{32}P-labeled complexes, halted at +24 (by privation of CTP from the initiation mixture), were immobilized and purified on streptavidin-coated magnetic beads. They were then incubated with the Rho variants indicated above gel lanes before being ‘chased’ with 75 µM rNTPs. The supernatant (*S*) and bead (*B*) fractions were then separated on a Magrack (GE-healthcare) before analysis by denaturing PAGE. The presence of the Rho-dependent transcripts in supernatants confirms that they stem from termination rather than transcriptional pausing or arrest. **(A)** Comparison of the RNA release efficiencies of the various Rho proteins. **(B)** Effect of the presence of BCM (0 or 750 µM) in reactions performed with the representative WT MtbRho or the [Mtb:Ec]Rho chimera.

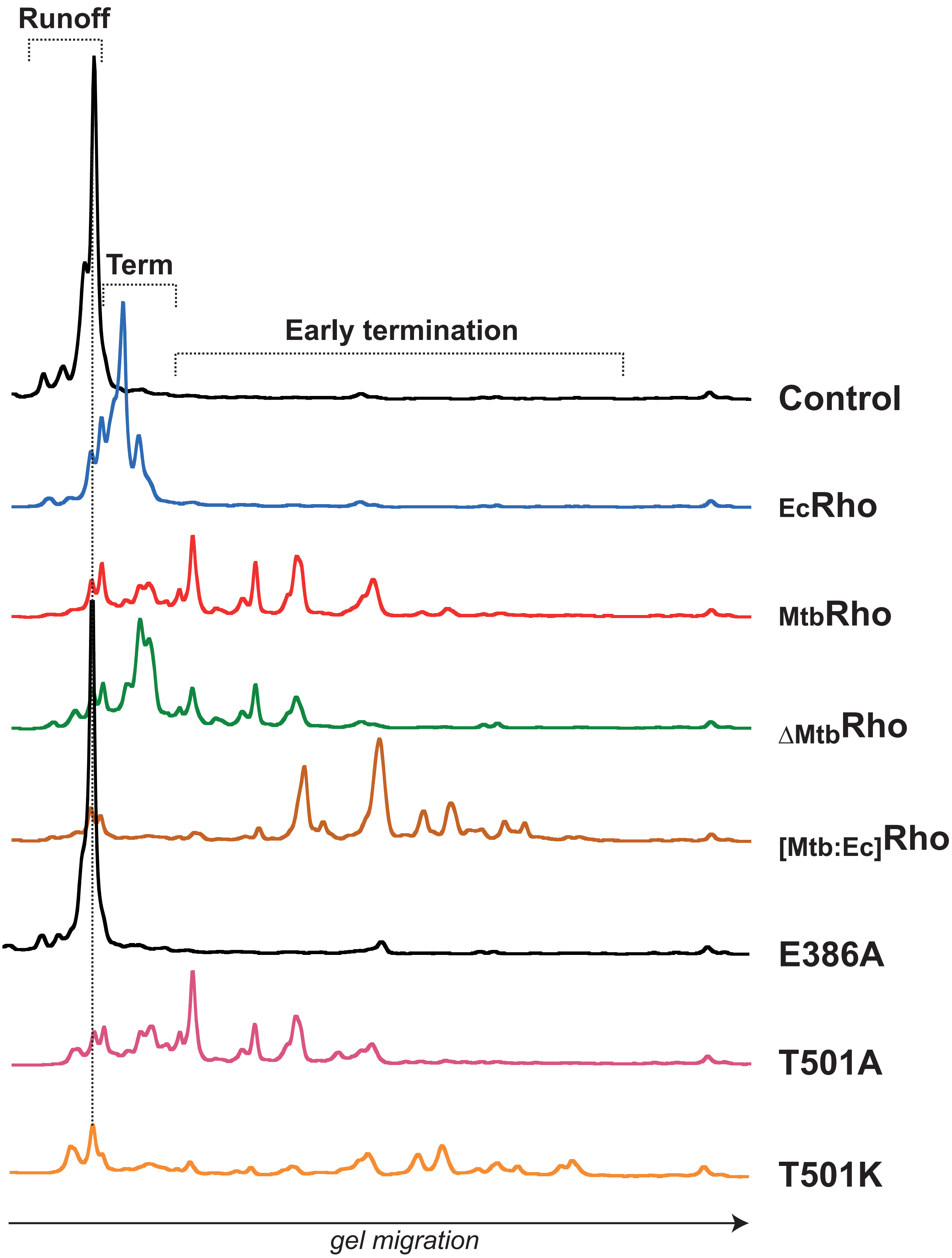

Supplementary figure 5: Normalized electrophoregrams of transcription termination reactions performed under standard ‘chase’ conditions (as in Figure 6B).

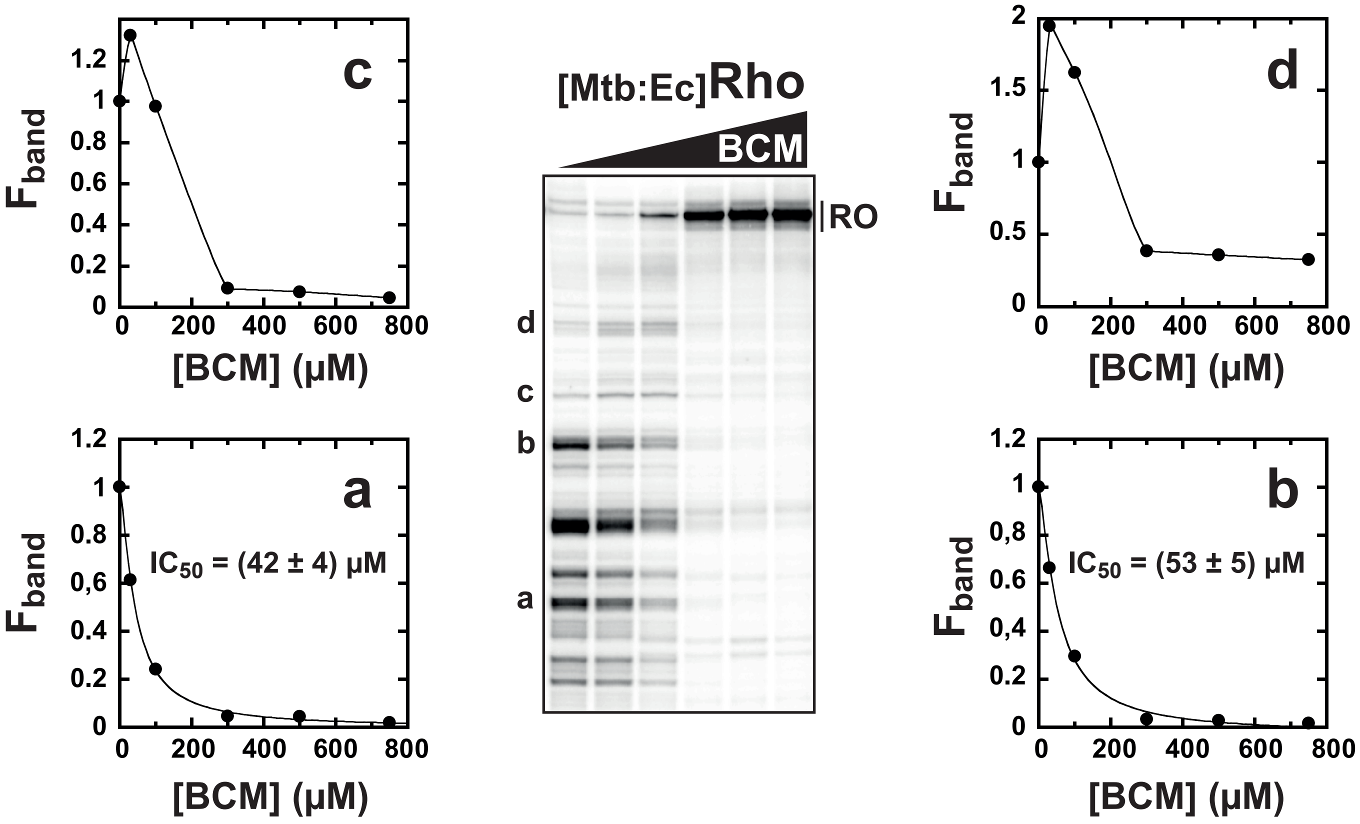

Supplementary figure 6: Upstream shift of the termination window obtained with the $_{[Mtb:Ec]}$Rho chimera as a function of BCM concentration. Graphs show normalized fractions of products released at representative termination positions as a function of BCM concentration (data averaged from 3 independent experiments).

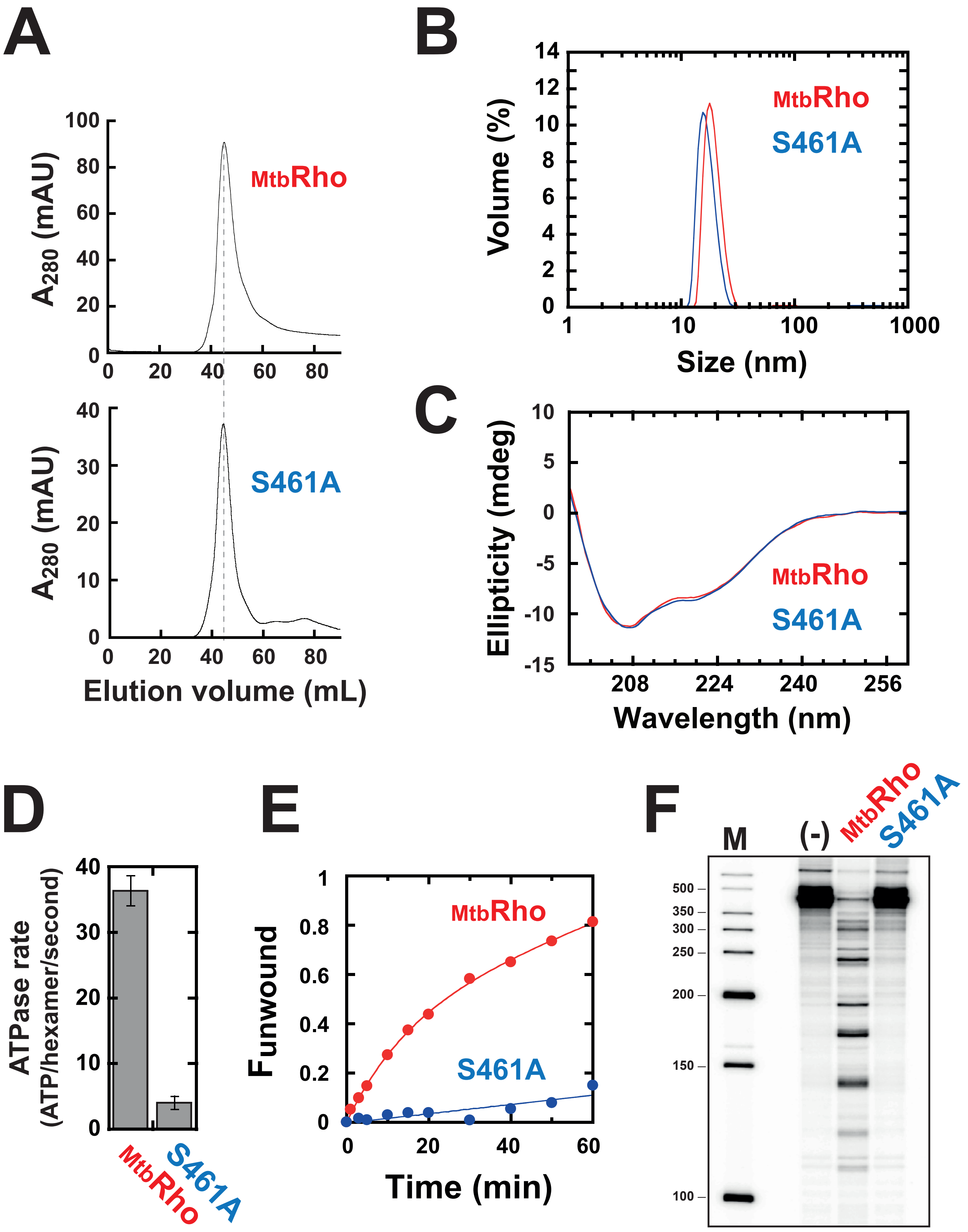

Supplementary figure 7: Characterization of the single-point mutant S461A of $_{Mtb}$Rho by **(A)** gel filtration on a Sephacryl S-300 HR column, **(B)** diffusion light scattering, **(C)** circular dichroism, and **(D)** steady-state ATP hydrolysis measurements. The Q-loop mutation greatly alters the capacity of $_{Mtb}$Rho to unwind duplex C **(E)** or to trigger transcription termination **(F)**. Data for WT $_{Mtb}$Rho are shown for comparison.
